# Comparative outcomes of day 4 versus day 5 embryo transfers by fertilization method

**DOI:** 10.3389/fendo.2026.1740765

**Published:** 2026-03-16

**Authors:** Lin-Lin Tao, Bo Zheng, Guo-Zhen Li, Yasong Geng, Zhi-Wei Yang, Hao-Yang Dai, Jing Ma, Fang-Fang Dai

**Affiliations:** 1 Reproductive Center, Xingtai Meihe Reproductive and Genetic Hospital, Xingtai, China; 2 Hebei Key Laboratory of Reproductive Medicine, Hebei Reproductive Health Hospital, Shijiazhuang, Hebei, China

**Keywords:** clinical outcomes, day 4 transfer, day 5 transfer, fertilization method, neonatal outcomes

## Abstract

**Objective:**

The aim of this retrospective cohort study was to evaluate the clinical and neonatal outcomes between day 4 and day 5 embryo transfer across different fertilization methods.

**Methods:**

This retrospective cohort study was conducted between January 2018 and March 2023, enrolling a total of 1,245 fresh embryo transfer cycles, including 1,023 *in vitro* fertilization (IVF) cycles and 222 intracytoplasmic sperm injection (ICSI) cycles. Among these, IVF cycles included 793 day 4 transfers and 230 day 5 transfers; ICSI cycles included 179 day 4 transfers and 43 day 5 transfers. The study conducted a comparative analysis of clinical pregnancy outcomes between day 4 and day 5 transfers.

**Results:**

In IVF or ICSI cycles, no significant differences were found in clinical pregnancy rate (CPR), implantation rate (IR), live birth rate (LBR), or other clinical outcomes between transfers of day 4 and day 5 embryos (*P* > 0.05). In IVF cycles with single high-quality embryo transfer, the CPR (62.72%, *P* = 0.026), gestational week of delivery (39 weeks, *P* = 0.026) of day 4 were significantly higher than day 5 (38 weeks). After controlling for potential confounding factors, the CPR of the day 4 group was also higher than day 5 (OR 0.578, 95% CI 0.352-0.949, *P* = 0.030). In IVF or ICSI cycles with day 4 high-quality embryo transfer, the LBR (63.06%, *P* = 0.006; 70.37%, *P* = 0.006) and multiple pregnancy rate (MPR; 53.29%, *P* < 0.001; 50%, *P* < 0.001) of transferring double high-quality embryo were significantly higher than transferring single high-quality embryo (LBR: 50.87%, 44.23%; MPR: 0%, 3.23%).

**Conclusion:**

In IVF or ICSI cycles, day 4 embryo transfer is considered a viable option or alternative to day 5 blastocyst transfer with no difference in clinical and neonatal outcomes. In IVF cycles with single high embryo transfer, day 4 transfer is recommended due to its significantly higher pregnancy rate compared to day 5 transfer. To reducing MPRs and preterm birth rates (PBRs), day 4 single embryo transfer is recommended if embryos achieve high-quality grade (full compaction) on day 4.

## Background

In the field of reproductive medicine, *in vitro* fertilization (IVF) is currently regarded as one of the most important treatments for infertility. Over the past few decades, IVF technology has made significant progress, yet a considerable number of patients still fail to achieve pregnancy. The method of fertilization, embryo quality, and timing of transfer are key factors influencing pregnancy success rates. Currently, many fertility clinics routinely perform day 3 and day 5 embryo transfer *in vitro* fertilization/intracytoplasmic sperm injection (IVF/ICSI) cycles. Multiple studies indicated that cleavage-stage embryo transfer had a lower clinical pregnancy rate (CPR) and higher miscarriage rate than blastocyst transfer (1, 2). This may be because blastocyst transfer allows for better synchronization between the endometrium and embryo growth, while also enabling the selection of higher-quality embryos (3, 4). However, other studies indicated that the cumulative pregnancy rate of blastocyst transfer was comparable to or even lower than that of cleavage-stage embryo transfer (5, 6).

Day 4 embryo transfer, however, is frequently disregarded. Compared with day 3, the silenced embryonic genome becomes active and the apoptotic system and cellular cycle checkpoints are activated (7). Hsieh et al. (8) demonstrated that day 4 embryos exhibited a higher rate of euploidy. On day 4 after fertilization, uterine contractions diminish, enhancing the receptivity of the endometrium to the embryo (9). Some studies indicated that the success rate of day 4 embryo transfer was significantly higher than that of day 3 transfer (10). In addition, under physiological conditions, the embryo enters the uterine cavity from the fallopian tube around the fourth day after fertilization. Therefore, transferring the embryo on day 4 more closely mimics the natural process. Furthermore, prolonged embryo culture leads to abnormal imprinted gene expression associated with apoptosis, oxidative stress and gap junction formation (11). Compared to day 5 transfer, day 4 transfer shortens the *in vitro* culture time, thereby reducing the risk of cycle cancellation due to prolonged culture duration. After day 4 embryo transfer, embryos that fail to develop into blastocysts may still develop within the uterine environment and successfully implant. In IVF/ICSI cycles that extend embryo culture to the blastocyst stage may reduce the number of embryos available for freezing and slightly increase the risk of adverse neonatal outcomes (5). Although, there is currently insufficient evidence to establish a causal relationship between the two. Furthermore, time-lapse imaging technology has now been widely adopted. This technology stabilizes environmental conditions, mitigates environmental impacts on embryos, and optimizes embryo selection processes through intelligent analysis of embryonic development, thereby reducing adverse effects of the environment on cultured blastocysts (12).

There are currently several studies on the pregnancy outcomes of day 4 and day 5 embryo transfers. Studies showed that the CPR and live birth rate (LBR) on day 4 of fresh IVF/ICSI cycles were similar to those on day 5 (13–16). Morula embryo transfer might also serve as an alternative option for clinicians in addition to cleavage stage and blastocyst stage (14–16). However, Alper et al. found that transfers of fresh embryos on day 5 were superior to those on day 4 and should be favored (17). Therefore, the superiority of day 4 versus day 5 embryo transfer in clinical outcomes remains unclear. Determining the optimal transfer timing could significantly improve implantation rates (IRs) and LBRs. Moreover, the impact of the fourth day on neonatal outcomes has rarely been reported. This study will investigate the comparison of pregnancy outcomes on day 4 and day 5, as well as the effects of day 4 transfer on newborns.

Different fertilization methods may lead to variations in fertilization and embryo outcomes. Simultaneously, the number and quality of embryos transferred are important factors influencing pregnancy outcomes. Therefore, stratified analysis of fertilization methods, the number and quality of embryos transferred is necessary. Previous studies (13, 16) have compared pregnancy outcomes between day 4 and day 5 embryo transfers, finding similar results. However, these analyses did not account for fertilization methods, embryo quality, or embryo quantity. Alper et al. (17) conducted a stratified analysis of embryo transfer numbers, but their study exclusively employed intracytoplasmic sperm injection (ICSI) fertilization techniques and did not stratify embryo quality. Moreover, their results contradicted previous studies, showing that day-5 embryos yielded superior pregnancy outcomes compared to day-4 embryos. This discrepancy may be attributable to the exclusive use of ICSI fertilization. Sun et al. (15) stratified embryo transfer numbers on day 4 and 5. No significant differences were observed between groups regardless of transferring 1 or 2 embryos. However, they did not perform stratified analysis based on fertilization method or embryo quality. These previous studies have not conducted stratified analyses for different fertilization methods, nor have they performed additional subgroup analyses based on embryo quantity and quality. Furthermore, neonatal outcomes are rarely reported. This study is the first to compare pregnancy and neonatal outcomes following day 4 and day 5 embryo transfers under different fertilization methods. Stratified analyses were also conducted based on the number and quality of embryos transferred. The advantages and disadvantages of various transfer strategies were examined, providing reference for selecting the timing of embryo transfer and developing individualized transfer protocols in assisted reproductive clinical practice.

## Materials and methods

### Study design and patients

This retrospective cohort study was conducted in the Reproductive Medicine Center of Xingtai Meihe Reproductive and Genetic Hospital. The study included patients who underwent a fresh transfer between January 2018 and March 2023, none of whom underwent preimplantation genetic testing (PGT). Exclusion criteria comprised cycles without embryo transfer, cycles with embryo transfer on day 2 or day 3, > 38 years old, endometriosis, genetic, metabolic diseases, congenital uterine abnormalities, other protocols besides gonadotrophin releasing hormone (GnRH) agonist pituitary down-regulation protocol as well as missing data in the electronic medical records.

**Figure 1:**
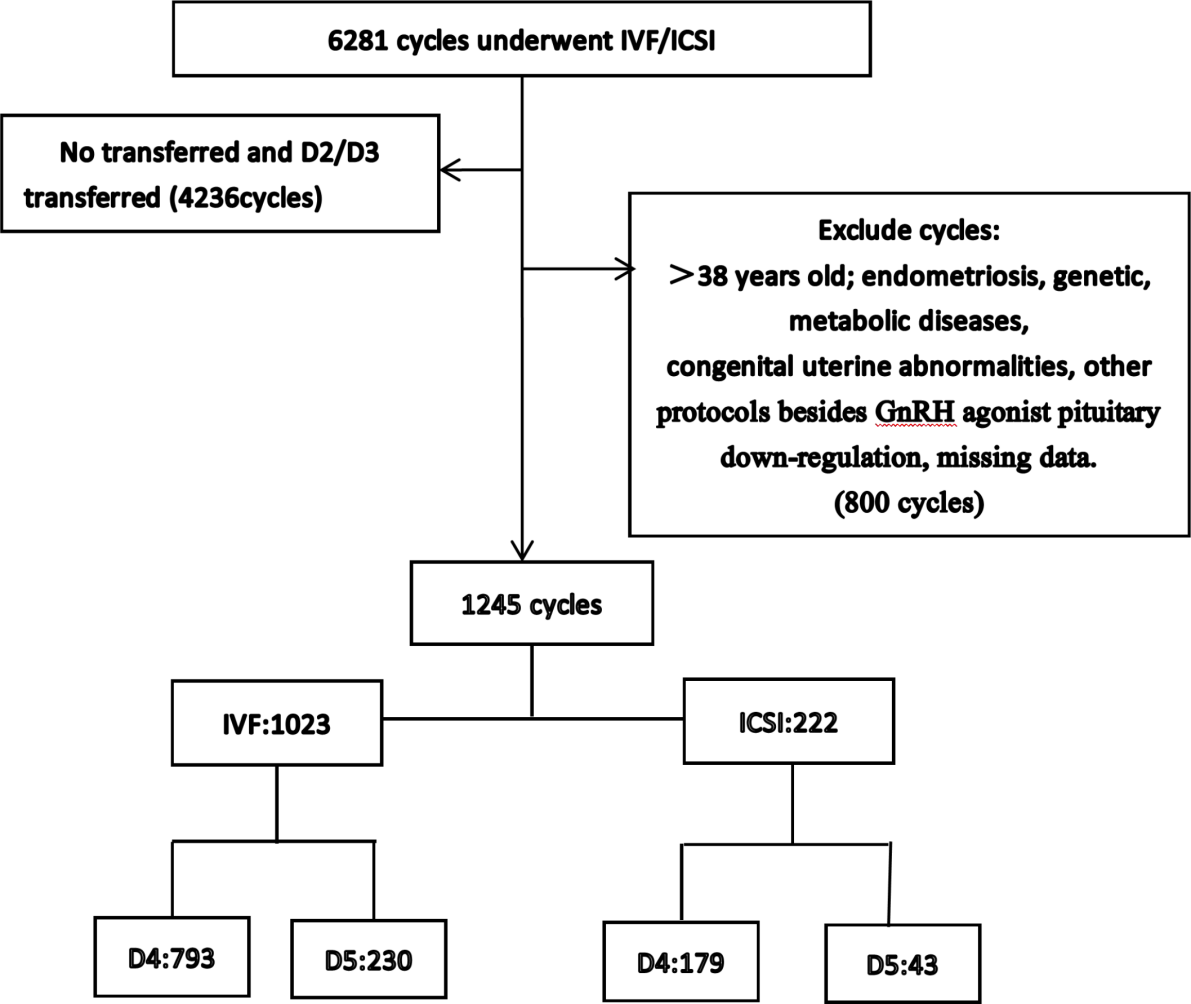
The flow chart of the study population.

This study included 1,245 fresh transfer cycles, including 1,023 IVF cycles and 222 ICSI cycles. Among these, IVF cycles included 793 day 4 transfers and 230 day 5 transfers; ICSI cycles included 179 day 4 transfers and 43 day 5 transfers (**Figure 1**). The study conducted a comparative analysis of clinical pregnancy outcomes between day 4 and day 5 transfers. This study was approved by the Ethics Committee of Xingtai Meihe Reproductive and Genetic Hospital (No. 2018-09). According to the Ethics Committee of Xingtai Meihe Reproductive and Genetic Hospital, the requirement for informed consent was waived.

### Ovarian stimulation

Pituitary down-regulation protocol using GnRH agonist was administered to all patients. Long-acting Duffelin (IPsen, France) in doses ranging from 1.0 to 3.75 mg was given. Then, 100–225 IU/d of recombinant follicle stimulating hormone (r-FSH, Precon, Merck, Netherlands) were given. Follicle growth was measured by measuring blood levels of progesterone (P), Luteinizing hormone (LH), and estradiol (E2), while follicle size was periodically measured by transvaginal ultrasonography. 6000–10,000 IU of human chorionic gonadotropin (HCG, Zhuhai Lizon Pharmaceutical) was injected when at least two leading follicles measured ≥ 18 mm. After 36–37 hours, the oocytes were retrieved out by vaginal puncture under intravenous anesthesia and ultrasound guidance.

### IVF procedures and assessment

Individual embryos were cultivated in microdroplets using Vitrolife’s G1-PLUS/G2-PLUS sequential media at 37°C in an incubator with saturated humidity, 6% CO_2_, 5% O_2_, and 89% N_2_. One or two embryos were transferred on day 4 or day 5. The 2011 ESHRE Istanbul Consensus (18) was used in our center to score day 4 embryos, while the Gardner scoring system (19) was used for blastocysts. A day 4 embryo that had lost all blastomere boundaries was termed a fully compacted embryo. Day 4 embryos with two pronuclear fertilizations, no vacuoles, and fully compacted (16) were defined as high-quality embryos. Blastocysts were scored in accordance with the Gardner blastocyst scoring system (19) on Day 5. In our laboratory, blastocysts were recorded as high-quality if they reached at least an expansion stage 3 with A or B for inner cell mass (ICM) and trophectoderm (TE). On days 5 and 6, any blastocysts that were not used for transfer would be cryopreserved. The standard for frozen blastocysts is that they reach stage 3 or above and that their ICM score is not C. These blastocysts would then be thawed and transferred once the patient’s physical condition and endometrial environment meet the required standards. Serum β-hCG levels were measured 12–14 days after embryo transfer to determine biochemical pregnancy occurrence. Approximately four weeks post-transfer, transvaginal ultrasound was used to visualize the gestational sac, cardiac tube, and fetal heartbeat. The number of gestational sacs was recorded, and the presence of a gestational sac was considered a clinical pregnancy. Luteal support continued until 12 weeks of gestation, followed by postpartum follow-up.

### Clinical outcomes

The primary outcome measure was clinical pregnancy rate. The secondary outcomes variables included rates of live birth, multiple pregnancies, ectopic pregnancy, miscarriage and cumulative live births, as well as neonatal outcomes. Neonatal outcomes included preterm birth, gestational age at delivery, proportion of males.

The clinical pregnancy rate was calculated by dividing the number of patients with at least 1 gestational sac detected by transvaginal ultrasound (performed 28 days after embryo transfer) by the number of patients transferred. A live birth was defined as a live baby delivered after 24 weeks of pregnancy. Multiple pregnancies were defined as the presence of multiple intrauterine fetuses simultaneously. Ectopic pregnancy was diagnosed using ultrasound or laparoscopic imaging of at least one ectopic pregnancy sac. Miscarriage was defined as the loss of fetal cardiac activity within 28 weeks of confirming clinical pregnancy. Preterm birth was defined as a birth before completing 37 weeks of gestation. Cumulative live births refer to the total number of live births achieved within a two-year period across all cycles following the current transfer, including the current fresh cycle and any subsequent frozen-thawed transfer cycles.

### Statistical analysis

All data were statistically analyzed using SPSS 22.0 for Windows (IBM, Armonk, NY, USA). The data was examined for normality. The average value of normally distributed measures was expressed as the mean ± standard deviation, and the T-Test for two independent samples was used to compare groups. Continuous variables that did not conform to a normal distribution were expressed as the median (25th, 75th percentile), M (Q1, Q3), and were compared using the Mann-Whitney U test. Categorical variables were expressed as frequencies and proportions and were compared using the chi-square or Fisher’s exact test, *P*-values < 0.05 were considered statistically significant. A prior power analysis was conducted using G*Power 3.1 with the following parameters: effect size w = 0.3, α err prob = 0.05, power (1-β err prob) = 0.80, and df = 1. The calculation yielded a minimum required sample size of 88 subjects. To investigate the effect of embryonic development days on CPR stratified by fertilization method, we performed multivariate logistic regression analyses. The female age, body mass index (BMI), basal FSH, anti-Müllerian hormone (AMH), type of infertility, infertility factors, E_2_ on the HCG day, LH on the HCG day, endometrium thickness, the number of retrieved oocytes, embryonic development days were used as independent variables, while the CPR was used as dependent variables in a logistic regression analysis. A significance level of *P* < 0.05 was considered statistically significant.

## Results

### Maternal and cycle characteristics

As shown in **Table 1**, comparing day 4 and day 5 embryo transfers across different fertilization methods, no significant differences were observed in baseline characteristics such as female age, BMI, duration of infertility, type of infertility, distribution of infertility factors, basal FSH, basal LH, AMH, total gonadotropins (Gn) doses, Gn duration, E_2_ on the HCG day, LH on the HCG day, P on the HCG day, endometrial thickness, the number of retrieved oocytes, embryos transferred number and number of high-quality embryos transferred.

**Table 1:** Baseline characteristics of the patients among day 4 and day 5 group stratified by fertilization method.

Variables	IVF		*P*	ICSI		*P*
	D4	D5		D4	D5	
Cycles(n)	793	230		179	43	
Female age [year, M (Q1, Q3)]	30.00 (28, 33)	30.00 (28, 33)	0.900	29.00 (26, 33)	29(27,33)	0.502
Female BMI [kg/m2, M (Q1, Q3)]	24.10 (21.60, 26.80)	24.20 (22.00, 26.60)	0.739	23.90 (21.90, 26.25)	23.9(21.70,26.40)	0.964
Infertility duration [years, M (Q1, Q3)]	3 (2, 5)	3.00 (2, 5)	0.512	4 (2, 6)	4(3,6)	0.151
Type of infertility (%)			0.691			0.528
Primary infertility	36.82 (292/793)	38.26 (88/230)		54.19 (97/179)	48.84 (21/43)	
Secondary infertility	63.18 (501/793)	61.74 (142/230)		45.81 (82/179)	51.16 (22/43)	
Proportion of infertility factors (%)			0.994			0.641
Unknown cause	1.77 (14/793)	1.74 (4/230)		–	–	
Ovulation disorder	12.74 (101/793)	12.17 (28/230)		11.73 (21/179)	18.60 (8/43)	
Male factor	1.51 (12/793)	1.30 (3/230)		58.66 (105/179)	55.81 (24/43)	
Fallopian tube factor	80.58 (639/793)	80.87 (186/230)		22.35 (40/179)	20.93 (9/43)	
Others	3.40 (27/793)	3.91 (9/230)		7.26 (13/179)	4.65 (2/43)	
Basal FSH [U/L, M (Q1, Q3)]	6.38 (5.36, 7.55)	6.33 (5.17, 7.57)	0.749	6.33 (5.29, 7.89)	6.95 (5.29, 7.96)	0.633
Basal LH [U/L, M (Q1, Q3)]	4.20 (3.00, 6.21)	4.20 (3.02, 6.77)	0.548	4.19 (3.17, 5.60)	5.08 (3.38, 6.21)	0.194
AMH [ng/ml, M (Q1, Q3)]	3.72 (2.53, 5.37)	3.71 (2.60, 5.43)	0.836	4.19 (2.70, 5.88)	3.76 (2.70, 5.31)	0.561
Total Gn doses [U, M (Q1, Q3)]	2450 (2025, 3000)	2475 (2025, 3000)	0.597	2475 (1950, 2925)	2425 (1988, 2875)	0.654
Gn duration [days, M (Q1, Q3)]	11.00 (10, 12)	12.00 (10, 13)	0.474	12.00 (11, 13)	12.00(11, 13)	0.837
E2 on the hCG day [pg/ml, M (Q1, Q3)]	2898(1912, 4081)	2892 (2000, 4086)	0.537	3349(2354, 4338)	3676 (2587, 4439)	0.470
LH on the hCG day [U/L, M (Q1, Q3)]	0.93 (0.69, 1.25)	0.89 (0.66, 1.20)	0.130	0.89 (0.74, 1.15)	0.98 (0.69, 1.15)	0.813
P on the hCG day [ng/ml, Mean ± SD]	0.77 ± 0.27	0.77 ± 0.27	0.859	0.79 ± 0.28	0.73 ± 0.22	0.224
Endometrium thickness [mm, Mean ± SD]	11.53 ± 2.23	11.46 ± 2.47	0.684	11.78 ± 2.21	11.99 ± 6.82	0.736
Number of oocytes retrieved [n, Mean ± SD]	13.52 ± 4.84	14.07 ± 4.86	0.133	13.91 ± 4.66	14.33 ± 4.42	0.597
Number of embryos transferred [n, M (Q1, Q3)]	2 (1, 2)	2 (1, 2)	0.079	2(1, 2)	2 (1, 2)	0.600
Number of high-quality embryos transferred [n, M (Q1, Q3)]	1(0, 2)	1 (1, 2)	0.874	1(0, 2)	1 (0, 2)	0.830

### Clinical outcomes and neonatal outcomes

**Table 2** provides a detailed overview of pregnancy outcomes between day 4 and day 5 embryo transfer cycles stratified by fertilization method. From these data, regardless of whether IVF or ICSI fertilization method, we found no significant differences in CPR, implantation rate (IR), LBR, miscarriage rate, multiple pregnancy rate (MPR), ectopic pregnancy rate, monozygotic twins rate and cumulative live birth rate (CLBR) between day 4 and day 5 embryo transfers. Neonatal outcomes, such as premature birth rate (PBR), stillbirth rate, cesarean section rate, gestational week of delivery, male/female ratio and live birth weight also showed no significant differences.

**Table 2:** The clinical outcomes of the patients in day 4 and day 5 group stratified by fertilization method.

Variables	IVF		*P*	ICSI		*P*
	D4	D5		D4	D5	
Cycles(n)	793	230		179	43	
Clinical Pregnancy rate (%)	63.93(507/793)	58.70(135/230)	0.148	65.36 (117/179)	65.12 (28/43)	0.976
Implantation rate (%)	51.09(655/1282)	50.98(182/357)	0.970	51.49 (155/303)	57.14 (39/71)	0.567
Live birth rate (%)	53.09(421/793)	48.70(112/230)	0.240	55.31 (99/179)	51.16 (22/43)	0.624
Ectopic pregnancy rate (%)	0.39(2/507)	0.74(1/135)	0.508	0.85 (1/117)	0(0/28)	1.000
Multiple pregnancy rate (%)	29.59(150/507)	34.81(47/135)	0.242	32.48 (38/117)	39.29 (11/28)	0.494
Monozygotic twins rate (%)	1.58(8/507)	1.48(2/135)	1.000	0.85 (1/117)	3.57 (1/28)	0.350
Miscarriage rate (%)	15.58(79/507)	16.30(22/135)	0.839	11.97 (14/117)	21.43 (6/28)	0.318
Premature birth rate (%)	16.17(82/507)	16.30(22/135)	0.973	14.53 (17/117)	7.14 (2/28)	0.466
Stillbirth rate (%)	0(0/507)	0(0/135)	/	0.85 (1/117)	0 (0/28)	1.000
Cesarean section rate (%)	57.99(294/507)	65.93(89/135)	0.095	58.97 (69/117)	67.86 (19/28)	0.387
Gestational week of delivery [weeks, M (Q1, Q3)]	38(37,39)	38(37,39)	0.067	38 (37,39)	38 (37,39)	0.584
Male/female ratio	0.99(267/273)	1.18(80/68)	0.320	0.97 (61/63)	1.31 (17/13)	0.463
Live birth weight [g, M (Q1, Q3)]	3000(2500, 3400)	2900(2550, 3388)	0.649	3200 (2600, 3500)	3000 (2663, 3188)	0.330
Cumulative live birth rate (%)	73.27 (581/793)	71.30 (164/230)	0.556	68.72 (123/179)	74.42 (32/43)	0.464

### Clinical and neonatal outcomes in subgroup analyses

**Tables 3**-**5** present a subgroup analysis evaluating the effect of the number and quality of embryos transferred on clinical outcomes. No further analysis was conducted for cycles involving the transfer of one high-quality and one non-high-quality embryo, as it was impossible to determine which embryo had successfully implanted. In addition, due to the limited number of single non-high-quality embryo transfers (IVF: 31 cycles, ICSI: 5 cycles), no further subgroup analysis was conducted. In IVF cycles with single high-quality embryo transfer, the CPR (62.72%, *P* = 0.026), gestational week of delivery (39 weeks, *P* = 0.026) of day 4 were significantly higher than day 5 (49.44%, 38 weeks) (**Table 3**). The LBR showed a trend toward increase but without significant difference. There was also no significant difference in CLBR. In IVF or ICSI cycles with double high-quality embryo transfer (**Table 4**), there were no significant difference in CPR, IR, LBR and CLBR between days 4 and 5 (*P* > 0.05). The same results were observed in IVF or ICSI cycles with double non-high-quality embryo transfer (**Table 5**).

**Table 3:** Outcomes of single high-quality embryo transfer on D4 and D5 stratified by fertilization method.

Variables	IVF		*P*	ICSI		*P*
	D4	D5		D4	D5	
Cycles(n)	287	89		52	13	
Clinical Pregnancy rate (%)	62.72 (180/287)	49.44 (44/89)	0.026	59.62 (31/52)	61.54 (8/13)	0.899
Implantation rate (%)	62.72 (180/287)	49.44 (44/89)	0.026	61.54 (32/52)	61.54 (8/13)	1.000
Live birth rate (%)	50.87 (146/287)	41.57 (37/89)	0.125	44.23 (23/52)	38.46(5/13)	0.707
Multiple pregnancy rate (%)	0 (0/180)	0 (0/44)	/	3.23 (1/31)	0(0/8)	0.607
premature birth rate (%)	5.56 (10/180)	4.55 (2/44)	1.000	9.68 (3/31)	12.51 (1/8)	1.000
Gestational week of delivery [g, M (Q1, Q3)]	39 (38, 40)	38 (38, 39)	0.026	38 (37, 39)	38(38, 39)	0.813
Cumulative live birth rate (%)	75.61 (217/287)	71.91 (64/89)	0.483	71.15 (37/52)	61.54 (8/13)	0.502

**Table 4:** Outcomes of double high-quality embryo transfer on D4 and D5 stratified by fertilization method.

Variables	IVF		*P*	ICSI		*P*
	D4	D5		D4	D5	
Cycles	222	63		54	14	
Clinical Pregnancy rate (%)	75.23 (167/222)	73.02 (46/63)	0.722	77.78 (42/54)	71.43 (10/14)	0.884
Implantation rate (%)	57.49(255/444)	59.85(76/126)	0.562	58.33 (63/108)	57.14 (16/28)	0.909
Live birth rate (%)	63.06 (140/222)	61.90 (39/63)	0.867	70.37 (38/54)	50 (7/14)	0.263
Multiple pregnancy rate (%)	53.29 (89/167)	65.22 (30/46)	0.149	50 (21/42)	60 (6/10)	0.828
premature birth rate (%)	26.95 (45/167)	21.74 (10/46)	0.475	21.43 (9/42)	10 (1/10)	0.706
Gestational week of delivery [g, M (Q1, Q3)]	38 (36, 39)	37 (37, 39)	0.750	38 (37, 39)	37 (37, 38)	0.464
Cumulative live birth rate (%)	76.58 (170/222)	84.13 (53/63)	0.200	77.78(42/54)	85.71 (12/14)	0.513

**Table 5:** Outcomes of double non-high-quality embryo transfer on D4 and D5 stratified by fertilization method.

Variables	IVF		*P*	ICSI		*P*
	D4	D5		D4	D5	
Cycles	188	42		52	11	
Clinical Pregnancy rate (%)	59.57 (112/188)	66.67(28/42)	0.395	51.92 (27/52)	72.73 (8/11)	0.354
Implantation rate (%)	39.94(266/666)	44.19(38/86)	0.450	33.04 (35/104)	54.17(12/22)	0.066
Live birth rate (%)	50 (94/188)	57.14 (24/42)	0.402	46.15 (24/52)	72.73 (8/11)	0.109
Multiple pregnancy rate (%)	35.71 (40/112)	35.71 (10/28)	1.000	29.63 (8/27)	50 (4/8)	0.402
premature birth rate (%)	14.29 (16/112)	21.43 (6/28)	0.523	14.81 (4/27)	0 (0/8)	0.553
Gestational week of delivery [g, M (Q1, Q3)]	38(37,39)	38(37,38)	0.127	39 (37, 39)	39 (38, 39)	0.405
Cumulative live birth rate (%)	70.21 (132/188)	71.43 (30/42)	0.876	57.69 (30/52)	81.82(9/11)	0.134

### Clinical outcomes according to high-quality embryo transfer number following different fertilization methods

**Figure 2:**
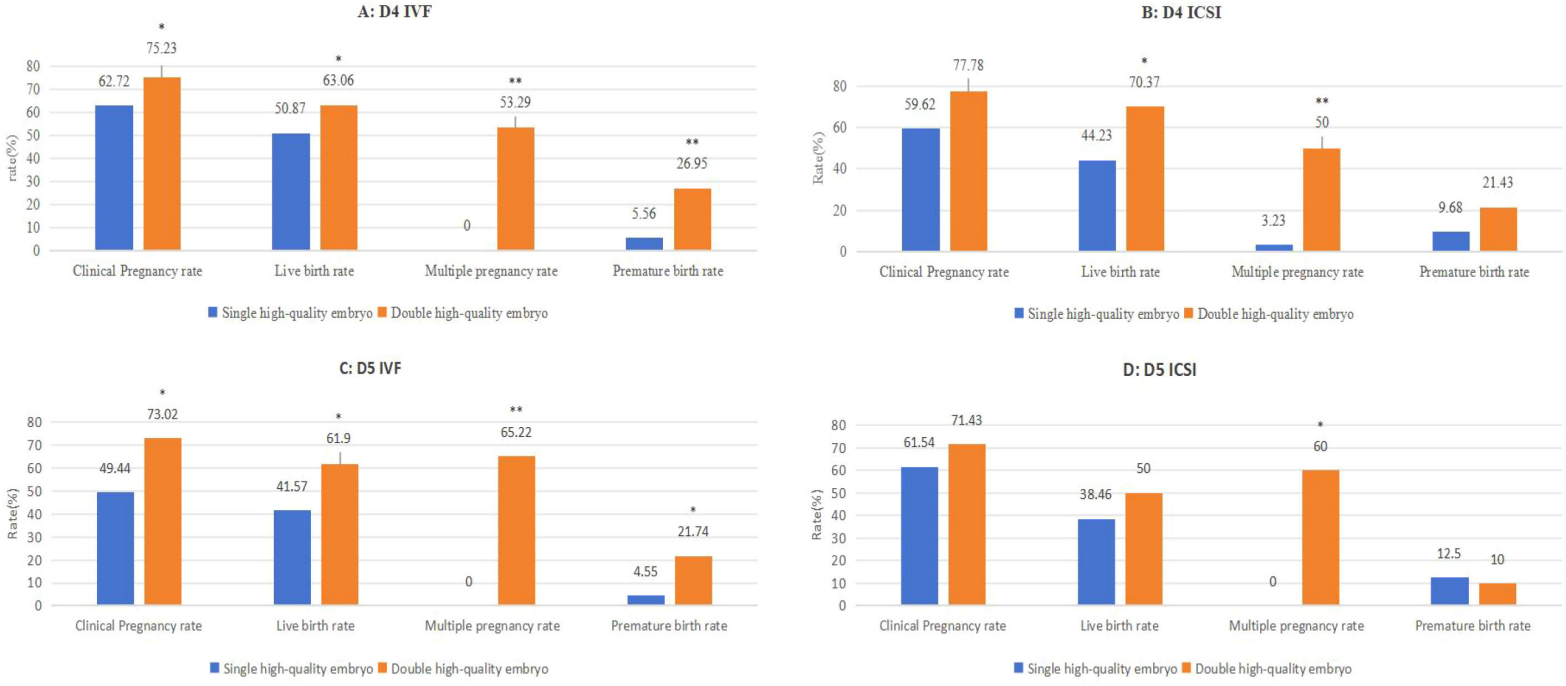
Clinical outcomes according to high-quality embryo transfer number on day 4 stratified by fertilization method “*” indicates P < 0.05 compared with transferring single high-quality embryo; “**” indicates P < 0.001 compared with transferring single high-quality embryo. **(A)** IVF cycle with high-embryo transfer on day 4. **(B)** ICSI cycle with high-embryo transfer on day 4. **(C)** IVF cycle with high-embryo transfer on day 5 **(D)** ICSI cycle with high-embryo transfer on day 5.

As shown in **Figure 2**, in IVF with day 4 or day 5 high-quality embryo transfer, the CPR (D4: 75.23%, *P* = 0.003;D5: 73.02%, *P* = 0.004), LBR (D4: 63.06%, *P* = 0.006; D5: 61.90%, *P* = 0.014), MPR (D4: 53.29%, *P* < 0.001; D5: 65.22%, *P* < 0.001) and PBR (D4: 26.95%, *P* < 0.001; D5: 21.74%; *P* = 0.016) of transferring double high-quality embryo were significantly higher than transferring single high-quality embryo [D4: (CPR: 62.72%; LBR: 50.87%; MPR: 0%; PBR: 5.56%); D5: (CPR: 49.44%; LBR: 41.57%; MPR: 0%; PBR: 4.55%). In addition, in ICSI cycles with day 4 high-quality embryo transfer, the LBR (70.37%, *P* = 0.006) and MPR (50%, *P* < 0.001) of transferring double high-quality embryo were significantly higher than transferring single high-quality embryo (LBR: 44.23%; MPR: 3.23%). In ICSI cycles with day 5 high-quality embryo transfer, the MPR (60%, *P* < 0.001) of transferring double high-quality embryo were significantly higher than transferring single high-quality embryo (0%).

### Logistic regression analysis on clinical pregnancy rate of D4 and D5 transfer stratified by fertilization method and embryos transferred number

As shown in **Table 6**, after controlling for potential confounding factors, in IVF cycles with single high-quality embryo transfer, the CPR of the day 4 group was higher than day 5 group (OR 0.578, 95% CI 0.352-0.949, *P* = 0.030).

**Table 6:** Logistic regression analysis of embryonic development days on clinical pregnancy rate in IVF or ICSI cycles.

Variables	IVF			ICSI		
	aOR	95%CI	P-value	aOR	95%CI	P-value
Single high-quality embryo transfer	0.578	0.352-0.949	0.030	1.168	0.249-5.477	0.844
Double high-quality embryo transfer	0.856	0.439~1.670	0.648	0.461	0.093~2.297	0.345
Double non-high-quality embryo transfer	1.317	0.633~2.738	0.462	1.438	0.251~8.247	0.684

## Discussion

In the present study, In IVF or ICSI cycles, no significant differences were found in CPR, IR, LBR, or other clinical outcomes between of day 4 and day 5 embryo transfers. In IVF cycles with single high-quality embryo transfer, the CPR, gestational week of delivery of day 4 were significantly higher than day 5. After controlling for potential confounding factors, the CPR of the day 4 group was also higher than day 5. In IVF cycles with double high-embryo transfer or double non-high-quality embryo transfer, clinical outcomes of day 4 and 5 were comparable. The same results were observed in ICSI cycles, but due to the limited sample size in the ICSI subgroup, further research is needed to validate these findings. Moreover, while transferring double high-quality embryo significantly increased the CPR and LBR, it also substantially elevated the MPR.

On the fourth day of embryonic development, cell numbers gradually increase and undergo compaction. During this process, the blastomeres progressively fuse, and the boundaries between them become increasingly indistinct. When the boundaries are nearly completely lost, the embryo appears as a single large cell-a stage known as complete compaction. Embryos developing more rapidly will form a blastocyst cavity. Embryonic compaction is essential for the formation of blastocyst trophoblast and inner cell mass (20). It has been reported that morula embryos with delayed and/or incomplete compaction have a reduced likelihood of developing into high quality blastocysts (21). In addition, the partial compaction group has more pronounced developmental delay at the post-fusion stage, which may affect blastocyst formation, implantation and live birth. Zhang et al. (14) demonstrated that full compaction and early blastocyst may result in higher pregnancy and live birth rates than partial compaction. Additionally, the 2025 Istanbul Consensus also recommends defining day 4 embryos with full compaction and no vacuoles as high-quality embryos. Therefore, in this study, day 4 embryos with two pronuclear fertilizations, no vacuoles, and fully compacted (16) were defined as high-quality embryos.

In the study, no significant differences were found in CPR, IR, LBR or other clinical outcomes between day 4 and day 5 embryo transfers in IVF or ICSI cycles. This finding is consistent with previous research (13–16). Therefore, day 4 embryo transfer is considered a viable option or alternative to day 5 blastocyst transfer. These findings collectively confirm the safety and efficacy of day-4 embryo transfer. Clinical institutions that have avoided day-4 transfer due to concerns about its outcomes may wish to consider adopting this approach. In addition, a day 4 transfer may be performed In clinical scenarios where day 3 or day 5 transfer is not feasible, including patient work schedules, non-standard laboratory hours, or scheduling constraints. Moreover, compared to morula culture, blastocyst culture is more complex and delicate, requiring a strictly controlled laboratory environment and technical support. For clinical institutions with limited technical capabilities, day 4 transfer may be a more suitable option.

To further investigate the applicability of Day 4 and Day 5 embryo transfer, we conducted a stratified analysis based on fertilization method, transferred embryo number and embryo quality. We found that in IVF cycles with single high-quality embryo transfer, the CPR, gestational week of delivery of day 4 were significantly higher than day 5. After controlling for potential confounding factors, the CPR of the day 4 group was also higher than day 5.Therefore, in IVF cycles suitable for SET, if high-quality embryos are available on day 4, performing a day 4 transfer may be a better option than a day 5 transfer. This result may be attributable to the reduction in the duration of *in vitro* embryo culture. Prolonged *in vitro* culture of embryos can lead to abnormalities in acquired modifications of genes (22), affecting embryo quality. In addition, it may also be related to endometrial factors. endometrial implantation window asynchrony or endometrial function defects lead to lower embryo implantation rate. For this group of individuals, the implantation window is most likely on day 4, resulting in superior pregnancy outcomes of day 4 transfers compared to day 5. The results of endometrial receptivity analysis (ERA) showed that delay or advancement of the endometrium in the implantation window by only 12–24 hours can affect implantation (23). However, there is no gold standard to demonstrate that individualized adjustment of hormone exposure time is enough to correct the non-receptive state endometrium, bridging the gap of ± (12-24) hours (23). Recent studies have reported changes in the transcriptome of uterine fluid-derived extracellular vesicles in response to changes in endometrial status. The trend of endometrial implantation window is consistent with the endometrial tissue transcriptome (24). It may be feasible to investigate the transcriptome of extracellular vesicles derived from uterine fluid as an alternative to RNA profiling of endometrial tissue. During embryo transfer cycles, assessing endometrial receptivity and performing the transfer during the optimal implantation window may improve implantation rates.

Nevertheless, in ICSI cycles with single high-quality embryo transfer, there was no significant difference in pregnancy outcomes between embryos transferred on day 4 and day 5. The cause may be related to inherent limitations in ICSI technology. This may be related to the inherent limitations of ICSI technology, which may increase the risk of conception using defective sperm (25). A study using sibling oocytes found that blastocyst formation rates were significantly lower in ICSI-derived embryos compared to IVF embryos. Consequently, ICSI may necessitate greater reliance on blastocyst culture to select embryos with superior developmental potential for transfer, thereby negating the advantage of day-4 transfer (26). But due to the limited sample size in the ICSI subgroup, further research is needed to validate these findings.

In this study, when transferring two high-quality embryos in IVF cycles, there was no significant difference between day 4 and day 5 transfer. Firstly, transferring two high-quality embryos increases the probability of embryo implantation and improves pregnancy outcomes compared to transferring one high-quality embryo. This effect mitigates differences associated with the day of embryo transfer. Secondly, transferring two high-quality embryos may produce a synergistic effect, leading to favorable pregnancy outcomes regardless of the transfer day or fertilization method. Research on embryos cultured *in vitro* indicates that pre-implantation embryos exhibit synergistic interactions *in vitro*, mediated by specific growth factors released by the embryos and highly dependent on embryo quality. Beyond embryo-to-embryo interactions, embryos also interact with the endometrium during the biological process of implantation. Recent studies indicated that signaling exchanges between embryos and the endometrium played a crucial role in embryo implantation (27–29). The endometrium, acting as a sensor for embryo quality, may recognize signals emitted by embryos of varying quality. Decidualized endometrial stromal cells (ESCs), serving as biomarkers for arrested embryos, can impede embryo implantation (30). A study examining the migration of women’s decidualized ESCs revealed significant differences in migration activity between high-quality embryos and low-quality embryos when placed on the surface of decidualized ESCs (30). Therefore, transferring two high-quality embryos together may be more conducive to implantation, thereby improving pregnancy rates. The outcomes of transferring two high-quality embryos during an ICSI cycle were comparable; however, due to the limited sample size in this subgroup, subsequent validation with a larger sample size is required. The results of this study also indicate that with day 4 or 5 transfer, transferring double high-quality embryo significantly improved the CPR, LBR compared to single high-quality embryo. However, this also led to a significant increase in MPR and PBR.

Multiple pregnancies have been reported to be associated with many obstetric and neonatal complications, such as hypertensive disorders of pregnancy, premature rupture of membranes, premature birth, postpartum hemorrhage and low birth weight babies (31, 32). The risk of preterm birth is six times higher in twin pregnancies than in singleton pregnancies, and the risk of low birth weight is ten times higher than in singleton pregnancies (33). Preterm birth can increase the risk of infant mortality and cause health problems, including long-term neurological defects (34). The most effective measure to reduce multiple pregnancies is to reduce the number of embryos transferred (35, 36). Therefore, it is recommended that single embryo transfer (SET) be performed in the presence of one or more high quality embryos on day 4 or 5. This is similar to the ASRM guidelines, which recommend SET in the presence of a euploid embryo is available (37). The study by Tighe et al. also demonstrated that two-consecutive single embryo transfer(2xSET) may provide greater or comparable live birth rates with lower multiple birth and morbidity than double embryo transfer (38). The 2xSET technique can be promoted according to maternal age and embryo quality in order to improve the reproductive outcome of IVF/ICSI pregnancies and reduce the risk of morbidity (38). However, when no high-quality embryos were available, transferring two non-high-quality embryos on day 4 did not yield better outcomes than transferring them on day 5. This is because embryos that fail to achieve full compaction on day 4, due to delayed densification, expansion, and cleavage expulsion, result in reduced blastocyst quality (39) and lower LBRs (40). This may have weakened the impact of day 4 transfer timing on pregnancy outcomes. Day 5 transfer allows for further embryo selection. Therefore, when no high-quality embryos are available on day 4, extending culture to day 5 for transfer may yield better pregnancy outcomes. The 2025 Istanbul Consensus also recommends extending partially compacted embryos to the blastocyst stage before clinical use (41). Additionally, in clinical practice, it remains necessary to develop personalized transfer protocols based on the patient’s age, history of previous embryo transfers, and other relevant factors to optimize pregnancy outcomes.

The strength of this study lies in its pioneering comparison of pregnancy and neonatal outcomes following day 4 versus day 5 embryo transfer under different fertilization methods. These findings may promote broader adoption of day 4 transfer techniques, particularly in resource-limited clinics or those traditionally inclined toward earlier transfers. A multi-level analysis was then conducted on embryo transfer quantity and quality, providing evidence-based guidance for selecting the timing and number of embryos to transfer, developing personalized transfer protocols, and optimizing maternal and fetal outcomes. However, this study had several limitations: First, it was a retrospective cohort study. The uneven distribution of transfer cycles on days 4 and 5 might introduce a certain degree of bias. Additionally, when considering day 4 or day 5 embryo transfer, physicians will comprehensively evaluate factors such as day 3 embryo quality and patient age. If conditions indicate favorable potential for blastocyst development, Day 5 transfer will be recommended. Although this study controlled for most baseline characteristics during logistic regression analysis, selection bias may still be present in retrospective studies and cannot be entirely eliminated; Second, this study was conducted at a single center. Differences in operational procedures, culture environments, and patient populations among *in vitro* fertilization centers in various regions may limit the generalizability of research findings. Third, at our fertility center, the number of ICSI cycles is relatively low. Subgroup analysis of embryo numbers had insufficient power due to small sample size. Fourth, this study did not analyze the outcomes of frozen embryo transfers and lacks relevant data. Therefore, the results of this study require further validation through prospective, multicenter clinical research with larger sample sizes in the future.

## Conclusion

In IVF or ICSI cycles, day 4 embryo transfer is considered a viable option or alternative to day 5 blastocyst transfer with no difference in clinical and neonatal outcomes. In IVF cycles with single high embryo transfer, the CPR of day 4 was significantly higher than day 5. Therefore, day 4 transfer is recommended. When performing double-embryo transfer, clinical outcomes of day 4 and 5 were comparable. However, transferring double high-quality embryo significantly increased the multiple pregnancy rate. To enhance CPRs while reducing MPRs and PBRs, SET is recommended if the embryos achieve high-quality grade on day 4 or day 5. In addition, there was no significant difference in pregnancy outcomes between transferring double non-high-quality embryos on day 4 versus day 5. If no high-quality embryo is available on day 4, it may be preferable to culture the embryos to the blastocyst stage before transfer. Additionally, in clinical practice, it remains necessary to develop personalized transfer protocols based on the patient’s specific situation to optimize pregnancy outcomes. Due to the retrospective, single-center nature of this study, certain limitations exist. In particular, the subgroup analysis of ICSI cycles had a small sample size, resulting in insufficient statistical power. Future prospective, multicenter clinical trials with larger sample sizes are required to validate these findings.

## Data Availability

The original contributions presented in the study are included in the article/supplementary material. Further inquiries can be directed to the corresponding author.
